# The State of Dark Coniferous Forests on the East European Plain Due to Climate Change

**DOI:** 10.3390/life12111874

**Published:** 2022-11-14

**Authors:** Konstantin E. Vedernikov, Irina L. Bukharina, Denis N. Udalov, Anna S. Pashkova, Maxim V. Larionov, Svetlana E. Mazina, Adelina R. Galieva

**Affiliations:** 1 Institute of Civil Protection, Department of Environmental Engineering Udmurt State University, Universitetskaya Street, 426034 Izhevsk, Russia; 2 Ministry of Natural Resources and Environmental Protection of the Udmurt Republic, 73 Maxim Gorky Street, 426051 Izhevsk, Russia; 3 World-Class Scientific Center “Agrotechnologies for the Future” (CAAT), Russian State Agrarian University—Moscow Timiryazev Agricultural Academy, 49 Timiryazevskaya Street, 127550 Moscow, Russia; 4 Faculty of Ecology and Environmental Protection, Russian State Social University (RSSU), 4 Wilhelm Peak Street, Building 1, 129226 Moscow, Russia; 5 Department of Economics and Management in the Fuel and Energy Complex, Institute of Industry Management, State University of Management (SUM), 99 Ryazanskij Prospect Street, 109542 Moscow, Russia; 6 Department of Digital Farming and Landscape Design, Faculty of Land Management and Environmental Management, Federal State Budgetary Educational Institution of Higher Education, “State University of Land Use Planning” (SULUP), 15 Kazakov Street, 105064 Moscow, Russia; 7 Research and Technical Centre of Radiation-Chemical Safety and Hygiene FMBA of Russian Federation, 40 Schukinskaya Street, 123182 Moscow, Russia; 8 Department of Environmental Safety and Product Quality Management for Educational Activities, Institute of Environmental Engineering, Peoples’ Friendship University of Russia (RUDN University), 6 Miklukho-Maklaya Street, 117198 Moscow, Russia

**Keywords:** spruce forests, climate change, unstable precipitation, temperature rise, change in the boundaries of natural zones, sanitary condition of spruce forests, bark beetles spread

## Abstract

As a result of global climate changes, negative processes have been recorded in the coniferous forests of the Northern Hemisphere. Similar processes are observed in the Urals, including in Udmurtia. In the course of this research, archival analysis methods were used, as well as field research methods. In the process of analyzing archival materials in the Urals, a reduction of spruce forests was observed. If in the 20th century the share of spruce forests in the region was 50%, then in the 21th century it decreased to 35%. As a result of this research, it was revealed that the most unfavorable sanitary condition was recorded in the boreal–subboreal zone of Udmurtia, with a sanitary condition index of 3.2 (from 2.62 to 3.73). The main reason for the unfavorable sanitary condition of spruce forests was the vital activity of *Ips typographus* L. According to our research, in 11 sample plots out of 18, a high score for sanitary condition was associated with the vital activity of bark beetles. The correlation coefficient of the index of the sanitary condition of plantings and the number of individuals of *Picea obovata* Ledeb. affected by *Ips typographus* L. was0.93. Bark beetle activity has increased in the 21th century, which is associated with changing climatic factors. Unstable precipitation over recent years (differences of more than 100 mm) and an average temperature increase of 1.2 °C were observed in the region. The most significant increase in temperature over the past 10 years was observed in winter, which in turn affected the high survival rate of insect pests.

## 1. Introduction

The Russian Federation has extensive forest resources, which play an important, and possibly decisive, role in atmospheric decarbonization, being a necessary element of the sustainable development of the planet [1]. Currently, it is necessary to pay special attention to the role of the Russian forest sector in adapting to climate change and mitigating its consequences, and it can play a decisive role in the global climate agenda. At the same time, the influence of climate change on forest ecosystems, especially on forests formed of *Picea obovata* Ledeb. (spruce forests) is also an important fact. The main reason for the reduction of spruce–fir forests in the 19th and 20th centuries was their intensive deforestation [2,3,4]. At the end of the twentieth century, mass drying of spruce forests in the Russian Federation began to be observed simultaneously in various regions of the European part of Russia. According to scientific research and official services at the end of the 1990s, the area of dead plantings increased by six times in comparison with the 1970s. The main causes (95% of total area of dead plantings) were the influence of adverse weather conditions, in which forest pests develop [5,6].

Researchers have noted that the unfavorable sanitary condition of forest cover is due to a decrease in the stability of forest ecosystems, against the background of global climate change. On the European continent, this process has manifested itself over huge areas and covered more than 10 tree species. An increase in the area of shrinking coniferous forests (pine, spruce, larch) has also been observed in Eastern European countries [7,8,9,10,11]. A forest, as a dynamic ecosystem, can adapt to gradually changing climatic conditions, but extreme weather changes lead to a loss of stability [12,13,14,15]. The expected global climate changes will change both the frequency and intensity of extreme weather events [16,17,18,19].

The trend of changing climatic factors in Russia began to be recorded after 1976. The warmest year was 2007, when the average annual temperature exceeded the climatic norm of 1961–1990 by 2.1 °C. The average linear trend of temperature increase from 1976 to 2012 was 0.043 °C/year. If one considers the entire territory of Russia, the temperature has risen most intensively (0.052 °C/year) in the European part of Russia [20], where dark coniferous forests are concentrated. An increase in the temperature regime has a beneficial effect on insects, especially pests, which began to expand their area, and in places of traditional habitat an outbreak of mass reproduction commenced.

There is a certain set of climatic indicators characteristic of the normal growth and development of tree species. A shift of these indicators beyond certain boundary values affects the area of plants, the composition of communities, and the sanitary condition of stands.

The objective of this work was to study the influence of climatic changes in the Urals on the condition of spruce forests.

## 2. Materials and Methods

**Figure 1:**
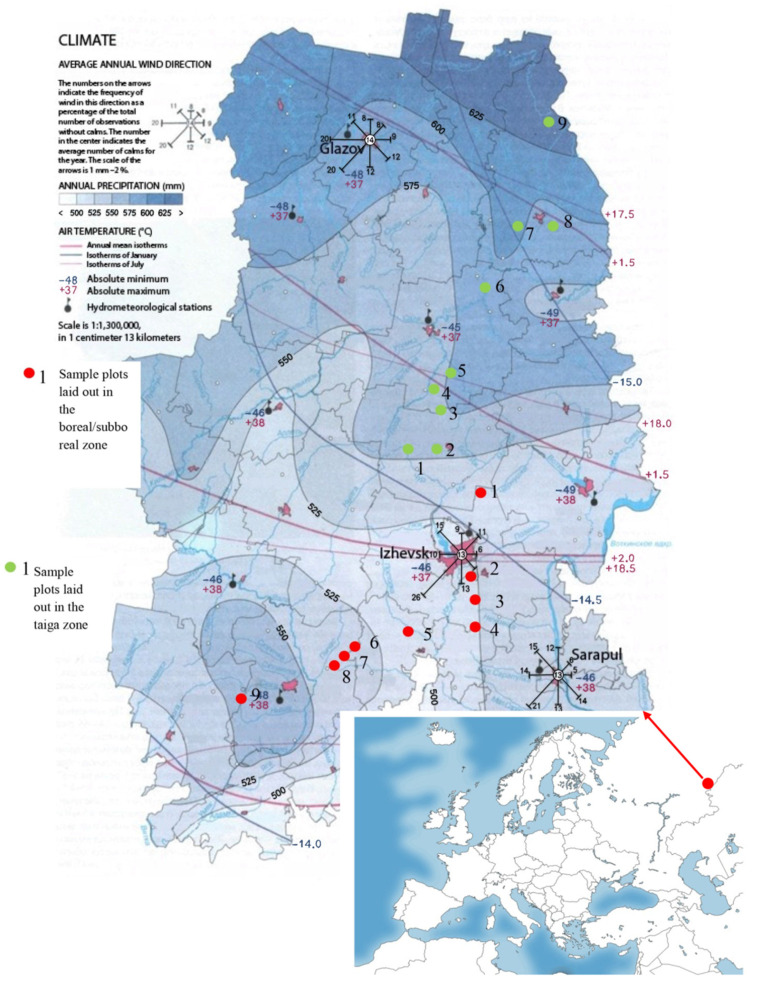
Features of climatic phenomena in the territory of the Udmurt Republic.

*Study area.* Research on climatic change and its influence on the condition of spruce forests was conducted in the Urals (in the territory of the Udmurt Republic). The Udmurt Republic is located to the west of the Ural Mountains, in the European part of the Russian Federation, and is a typical region (with some features) of the Pre-Urals. The climate within the territory of Udmurtia varies greatly, both in temperature and humidity. Data on the diversity of climatic factors in the research region are presented in Figure 1. In this regard, the territory of the republic is divided into two natural zones: the southern part of the republic is boreal–subboreal, and the northern part is taiga [21]. Study of the dynamics of weather phenomena was carried out separately in the southern and northern part, on the basis of official data. The research period was from 2009 to 2020. http://www.pogodaiklimat.ru/history/28315.htm (accessed on 12 June 2022).

*Materials and methods.* The influence of climatic factors on indigenous vegetation was studied using the example of spruce stands in the region. An analysis of the dynamics in the areas of spruce forest was carried out on the basis of reporting materials from regulatory authorities in the field of forestry, covering the period from 1965 to 2019 (materials of the state forest register of the Ministry of Natural Resources and Environmental Protection of the Udmurt Republic; Forest Plan of the Udmurt Republic, 2008; 2019; Forestry Regulations of 25 forestries, 2008; 2019; inventory materials of forests 1965; 1993–1997; 2016).

To assess the condition of spruce forests, sample plots of 100×100 m were laid, in which each tree was evaluated [22]. Sample plots were located in natural zones: boreal–subboreal (southern part of the republic), taiga (northern part of the republic).The locations for sample plots was selected in advance, based on the study of materials from forest departments. Sample plots were located in the territory of the studied region from north-west to south-east. The main areas of spruce forests are concentrated in this direction. Sample plots had to meet the following criteria: they should be located in areas with a high proportion of coniferous forests and should experience minimal economic impact (protected forests, where clear cutting is prohibited). The forest areas had to be homogeneous in age (average age) and in the species composition of the tree layer (70–100% of *Picea obovata* Ledeb.). The type of forest ecosystem was the same in all test areas (Table 1).

The condition of the trees was determined by external morphological features, following the scale of categories of tree condition presented in Table 2, including infection by pathogenic organisms (this scale is recommended in the Regulation of the Government of the Russian Federation for forest pathology assessment in Russia).

Each category of sanitary condition was assigned a score following the scale presented above. Then the weighted average score of the sanitary condition of the stand was determined using the formula:

Save. = (*P*1 × *K*1 + *P*2 × *K*2 + *P*3 × *K*3 + *P*4 × *K*4 + *P*5 × *K*5)/100 (1)

where Save.—weighted average score of the stand sanitary condition;

*P*i—share of spruce individuals in each condition category, %;
*K*i—index of tree state category (data from Table 1).

The sanitary condition of the stand was assessed usingthe weighted average score of the sanitary condition following the scale: 1–1.5—forest stands without signs of weakening; 1.51–2.5—weakened forest stands; 2.51–3.5—severely weakened forest stands; 3.51–4.5—drying forest plantations; more than 4.5—dead forest stands.

The results obtained were processed using mathematical data processing methods, using the Statistica 6.0 statistical software package (descriptive statistics method, correlation analysis).

## 3. Results

According to natural and climatic conditions, the Urals are favorable for the growth of spruce plantations. To maintain the stable condition and productivity of spruce forests, the following environmental conditions are necessary: average annual air temperatures from −2.9 to +4 °C; average temperature of the warmest month (July) from +10 to +20 °C; duration of the vegetation period, 110–175 days; the sum of temperatures above 5 °C in the range from 700 to 2230 °C, above 10 °C, from 500 to 1850 °C; and precipitation, 400–850 mm [23].

The spruce forests of Udmurtia are formed by *Picea obovata* Ledeb. stands, with the addition of hardwoods and Siberian fir (*Abies sibirica* Ledeb.). Spruce stands are concentrated in the northern part of Udmurtia, in the taiga zone (74% or 587,027 ha), while in the south, in the boreal–subboreal zone, the area of spruce stands is 204,009 ha or 26%. The spruce stands of Udmurtia are high-performance plantations, formed on sod-podzolic soils rich in mineral elements, with sufficient moisture [24,25].

**Figure 2:**
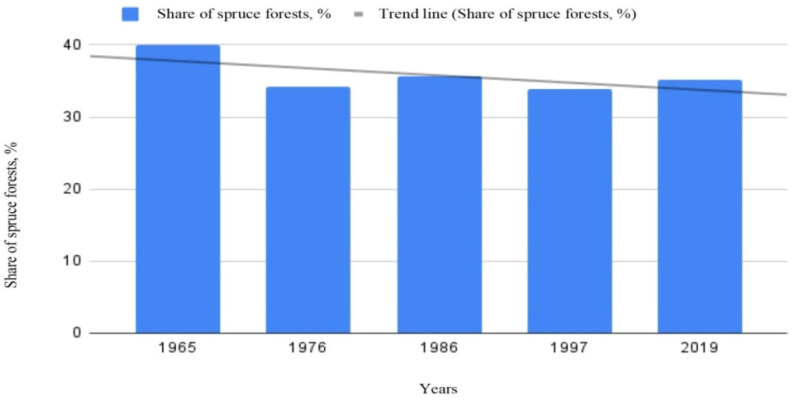
The share of *Picea obovata* Ledeb. stands on the territory of the East European Plain (with the example of the Urals), % (these data were calculated by the authors based on the analysis of the reporting materials of forest departments of the Udmurt Republic).

According to inventory data, a decrease in the proportion of spruce forests and an increase in deciduous forests has been observed in Udmurtia. According to the inventory data in 1965, the area of spruce stands was 40% of the total forest area, whereas at present (data for 2019), spruce stands make up only 35.2% [26]. Data on the change in the area of spruce forests inUdmurtia are presented in Figure 2.

The reduction in the area of spruce stands was observed against the background of an increase in the total area of forests in Udmurtia and an increase in the share of deciduous stands. Over the past 10 years, there has been a slight increase in the proportion of birch forests, associated with the displacement of spruce stands by silver birch (*Betula pendula* Roth.). The share of birch forests in Udmurtia increased by 1%, despite the fact that 100% of the forest is planted with conifers, and birch reproduction is not carried out in the territory of Udmurtia [26].

**Figure 3:**
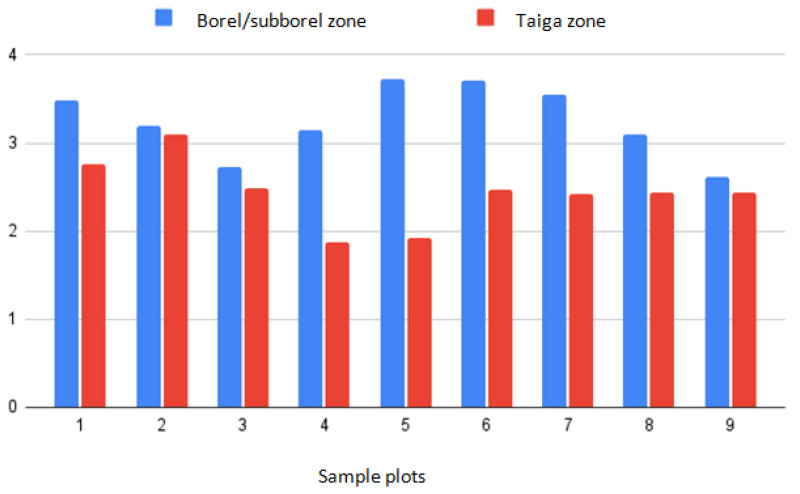
Index of the sanitary condition of spruce plantations in Udmurtia.

There was not only a reduction in spruce forests, but also a deterioration in their sanitary condition. According to our research, in general, the sanitary condition decreased in the boreal–subboreal and the taiga zones. The sanitary condition index ranged from 1.87 to 3.73 (boreal–subboreal zone 3.25 ± 0.38; taiga zone 2.41 ± 0.36) (Figure 3).

Unfavorable sanitary conditions could be associated with climate change. 

As confirmed by the latest scientific data, global climate change determines climatic characteristics from temperature to humidity. At the same time, regional changes may differ from the global anomalies. In Russia, the average annual temperature increase (1.6 °C) is almost an order of magnitude greater than the global one (0.9 °C) [27,28].

According to hydro meteorological observations, significant climate change in Udmurtia has been observed in the last 10–15 years. From 2006 to 2015, there was a decrease in precipitation during the vegetation period, with an increase in temperature (by an average of 1.2 °C) [28].

**Figure 4:**
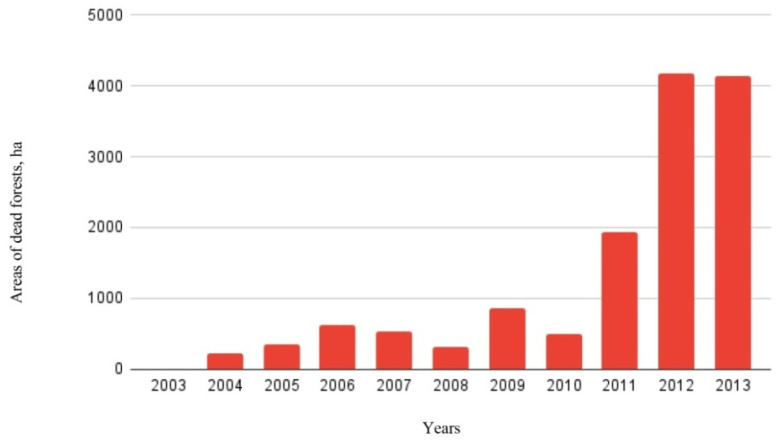
Variability of the areas of dead stands of *Picea obovata* Ledeb. in the forest ecosystems of the East European Plain (on the example of the Urals) [30].

According to in the Forest Protection Center, a massive reduction in the spruce forests in the region has been observed in the last 10 years, and this is associated with unfavorable climatic phenomena. Insufficient precipitation and intense waves of drought have led to the weakening of the protective mechanisms of spruce, which, as a consequence, has led to the massive development of *Ips typographus* L. [29,30]. The dynamics of dead forest stands in Udmurtia in 2002–2013 are shown in Figure 4.

## 4. Discussion

According to the Udmurtia Forest Plan, only 66% of the total potential annual volume of wood is harvested; in particular, for spruce stands this is 46%. Reforestation activities in the territory of Udmurtia are carried out in full and, on average in the republic, amount to 102% of the planned activities. Of the total planting material used for forest restoration, 87.6% is spruce [29]. Thus, it can be argued with a high probability that the reduction of spruce stands in the region is not related to economic activity.

According to the sanitary and forest pathology review of the state of forests in the Udmurt Republic in 2013, the area of dead stands was three-times higher than the average annual figure for 10 years. This happened as a result of the impact of abnormally high temperatures during the vegetation period of 2010. As a result of this phenomenon, spruce stands lost their resistance to insects, especially to *Ips typographus* L., which led to their death [31,32,33].

The presence of a large fodder base and an average annual temperature increase (by 1.2 °C in Udmurtia over the past 10 years) affected bark beetle survival favorably, and cases of the incubation of several generations of *Ips typographus* L. during the vegetation period have also been recorded.

The active phase of bark beetle development began in the boreal–subboreal zone of the region, and we recorded the unfavorable sanitary condition of spruce forests in this very zone [34,35]. In this zone, the index of sanitary condition of stands ranged from 2.62 to 3.73 (average 3.25, the higher the index of sanitary value, the worse the sanitary condition of plantings). Plantings were characterized as severely weakened. In the taiga zone, the index of sanitary condition varied from 1.87 to 3.09 (average 2.41); thus, spruce stands in the northern part of Udmurtia were characterized as weakened.

The unfavorable sanitary condition of spruce stands in the boreal–subboreal zone is associated with the vital activity of *Ips typographus* L. In most of the studied stands in the southern part of Udmurtia, more than 50% of the total number of spruce trees were found to be old deadwood trees (category 5 of the sanitary condition in Table 2), with typical traces of bark beetle activity. The closeness of the relationship between the index of the sanitary condition of plantings and the number of individuals of *Picea obovata* Ledeb. affected by bark beetle was positive and extremely high (correlation coefficient 0.93 *p* = 0.03). The worse the sanitary condition of the plantation, the higher the percentage of spruce individuals affected by bark beetles.

In the twentieth century, scientists noted that bark beetles did not play a decisive role in the state of spruce forests in Russia, due to the high percentage of deaths in winter because of low temperatures [36,37,38]; this is typical for Udmurtia as well. In the archival data analysis, outbreaks of mass reproduction of *Ips typographus* L. have not been recorded in the territory of Udmurtia, although local foci have been observed periodically since 1956.

The beginning of mass reproduction outbreaks of *Ips typographus* L. in Udmurtia was associated with the drought in 2010, but further population development was due to the high survival rate of the insect. The main limiting factors for bark beetles are temperature factors: low temperatures (up to −30−–40 °C) in winter, affecting the survival of adult beetles, and hot temperatures at the beginning of summer (22–30 °C), causing high mortality rates of larvae [39].

**Figure 5:**
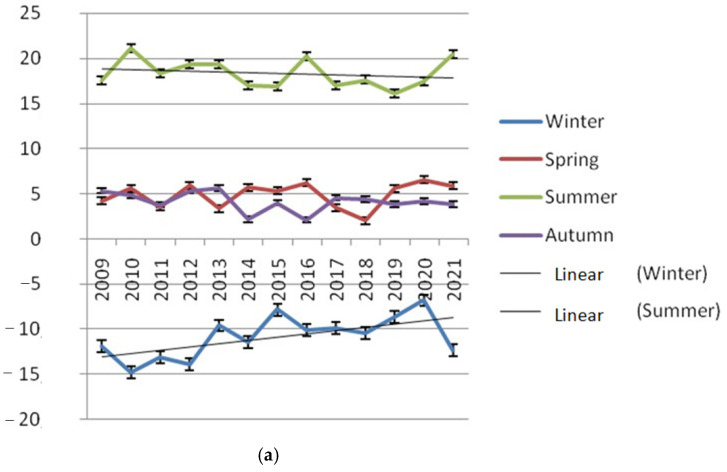

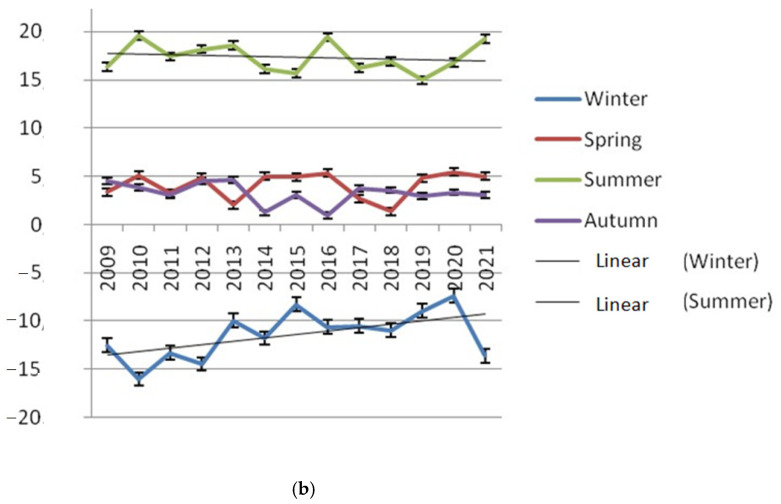
Average temperature by seasons in the Udmurt Republic, °C ((**a**)—boreal–subboreal zone, (**b**)—taiga zone) (the analysis was carried out by the authors using long-term data of the Russian Climate Monitoring Service).

In this regard, the mass reproduction outbreaks of bark beetle likely were caused precisely by favorable temperature factors in winter and summer. This is confirmed by the data presented in Figure 5. According to the analysis of temperature factors over the past 10 years, there has been a decrease in temperatures in summer and an increase in winter, in both the boreal–subboreal and taiga zones of Udmurtia. The most significant changes we rerecorded in winter.

**Figure 6:**
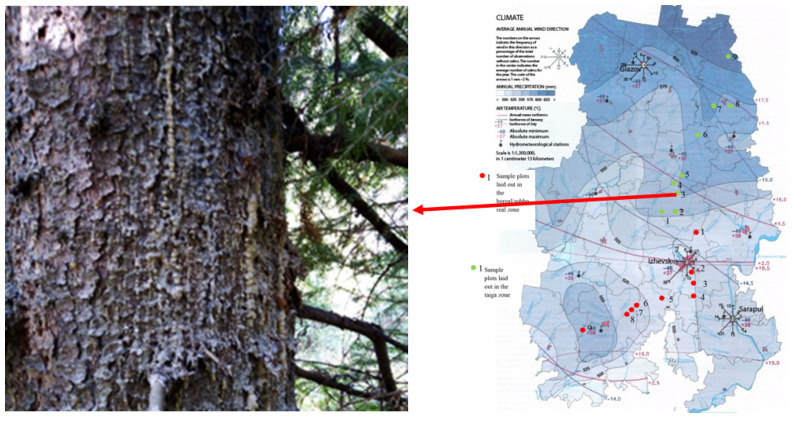
Butt infection of *Picea obovata* Ledeb. with bark beetles (sample plot No. 3 in the taiga zone).

In the territory of Udmurtia and in the European part of the Russian Federation, cases of several generations (up to 4 generations) of *Ips typographus* L have been recorded in spruce plantations during the vegetation period. This contributes to the expansion of the bark beetle area. If the focus of *Ips typographus* L. distribution in Udmurtia was in the boreal–subboreal zone, then during the study, we identified individuals of *Picea obovata* Ledeb. in the taiga zone, with a butt infection type in trees (Figure 6).

This type of infection is only noted in conditions of high population density of *Ips typographus* L. This indicates the expansion of the area pf mass reproduction outbreaks of the insect into the north of the studied region, which, in our opinion, is due to the improvement of climatic factors for bark beetle development.

*Ips typographus* L. inhabits weakened trees, in connection with which an outbreak of mass reproduction is possible in conditions of mass weakening of spruce individuals (for example, during windfall, drought, or in conditions of industrial pollution) [39,40]. Due to the fact that the spruce root system has a superficial location, the amount of precipitation is critical. Both alack and excess of moisture affects the condition of the plant negatively.

**Figure 7:**
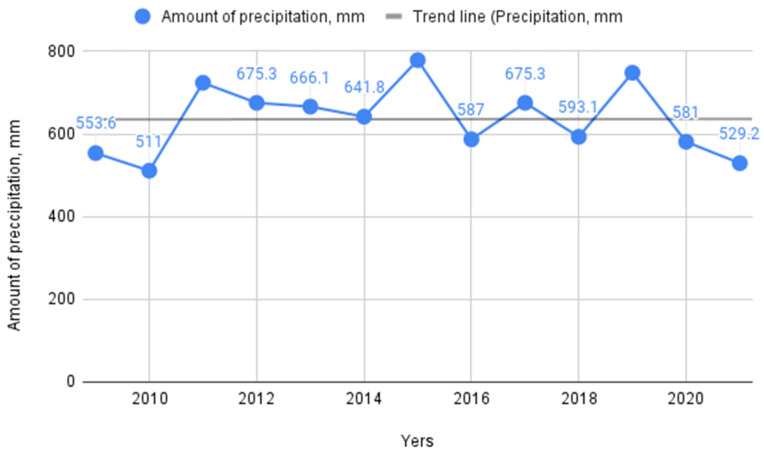
Average annual precipitation in Udmurtia, mm.

The analysis of precipitation data in Udmurtia as a whole for the analyzed period did not reveal any deviations; this shows that the amount of precipitation in the region has not changed over the past decade. Meanwhile, it should be noted that, depending on the year, the amount of precipitation differed by almost 100 mm, which indicates the instability of precipitation over the years, as shown in the Figure 7.

Unstable precipitation and an increase in the average annual temperature led to a shift to the north of the border of the boreal–subboreal zone in Udmurtia. Such climatic changes have a positive effect on the range of deciduous plant species, by expanding it, but at the same time displacing coniferous forests from indigenous places of growth.

**Figure 8:**
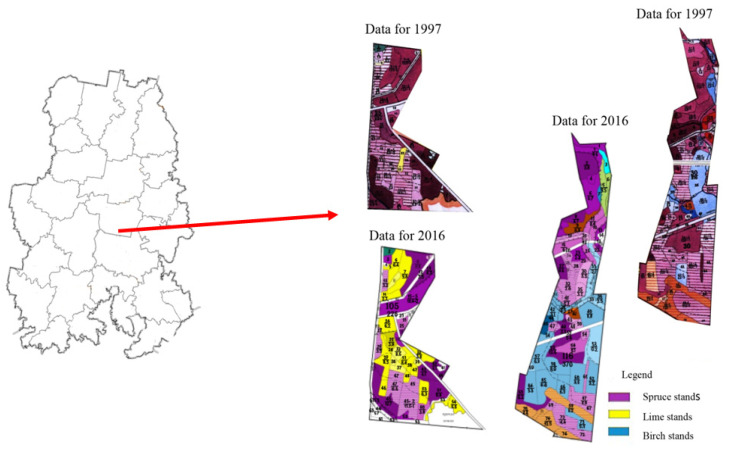
Change in the species composition of forests in Udmurtia (data from 1997 and 2016, with the example of one of the forest areas the in taiga zone). Map scale 1:35,000.

Thus, according to this study, in one of the forest areas of the taiga zone bordering the boreal–subboreal zone, a change of spruce forests to deciduous forests was recorded. For example, in the forest quarter No. 105 of Mukshinskii district forestry in 1997, the stock of spruce stands was 24,643 cubic meters of timber, and in 2016 this was 19,503.7 cubic meters of timber, which is 20.9% less. In forest quarter No. 116 of Mukshinskii district forestry, in 1997, the stock of spruce stands was 23,841.8 cubic meters of timber, and in 2016 this was 1917.4 cubic meters of timber, which is 19.6% less (Figure 8).

According to the species composition, the change of spruce stands to deciduous stands is obvious, with mainly small-leaved linden (*Tilia cordata* Mill.). It should be noted that in the taiga zone, small-leaved linden, due to unfavorable climatic factors, occupied the understory. This species could not enter the main canopy, much less form a full-fledged stand. However, the observed phenomenon fully reflects the trend of displacement of indigenous coniferous forests by deciduous forests as a result of the ongoing climatic changes.

## 5. Conclusions

Against the background of weather and climatic factors, limiting biotic factors in relation to forest stands can indeed appear [1,7,8,9,10,11,15,29,30,35,36,40].The performed analysis of scientific publications and official materials shows that the species areas of coniferous forest-forming tree species are quite sensitive to climate changes.

Monitoring the sanitary state of forest stands is the preferred approach in assessing the biological resistance of woody plants to limiting factors [22,41,42]. The data obtained by us reflect the change in the state and tolerance of forest stands against the background of weather and climate conditions in the territory of the implemented studies.

In the zone of boreal coniferous forests, including in the Urals, temperature is a limiting factor the vital activity of insect pests. An increase in temperature in winter and a decrease in summer makes the weather conditions optimal for the development of *Ips typographus* L. in the Urals. Taking into account the great fodder base (the share of spruce forests in Udmurtia is 33%) and favorable weather conditions, this allows this bark beetle to form several generations.

In conditions of unstable precipitation and the mass development of *Ips typographus* L., the spruce forests of Udmurtia are drying. These phenomena are most noticeable in the boreal–subboreal zone (sanitary condition index is 3.2). Although, in the taiga zone, plantings affected by bark beetles have also been identified. Thus, changes in climatic factors, through changes in the behavior of insect pests, affect the distribution and condition of spruce plantations.

During the 20th century spruce forests in Udmurtia were the dominant forest formation in50% of the total forest area [23]. Whereas, in the 21st century, 35.2% of them remained, despite active reforestation (80% of all planting material for reforestation is *Picea obovata* Ledeb.) [27].

The death of spruce forests is associated with the vital activity of bark beetles. According to our research data, 11 sample plots out of 18 have a high score for sanitary condition (unfavorable condition) associated with the vital activity of *Ips typographus* L. The correlation coefficient of the index of the sanitary condition of plantings and the number of individuals of *Picea obovata* Ledeb. affected by bark beetles is 0.93.

At the same time, it should be noted that the resistance of spruce individuals and the activity of bark beetles directly depend on the nature of climatic changes. The instability of precipitation by year (depending on the year, the difference in the amount of precipitation can be more than 100 mm) and the change in temperature regime promote greater survival of bark beetles.

## Figures and Tables

**Table 1:** Characteristics and coordinates of sample plots.

Number Sample Plots	Composition *	Coordinates
Boreal–subboreal zone		
1	9E1P	56.666018/53.323223
2	9E1P	56.722518/53.306079
3	9E1P	59.984736/53.424564
4	10E	56.599405/53.339568
5	10E	56.589050/52.941519
6	10E	56.505821/52.644072
7	9E1P	56.498542/52.594524
8	9E1P	56.506854/52.581571
9	9E1C	56.367733/52.075571
Taiga zone		
1	7E1P1B1Os	57.106117/53.066883
2	9E1Os	57.121483/53.158433
3	8E2P	57.281983/53.132300
4	8E2P	57.331900/53.160367
5	9E1P	57.377800/53.256750
6	8E2P	57.603850/53.456667
7	8E2P	57.848983/53.774517
8	9E1P	57.864967/53.694433
9	8E2P	56.198022/53.716683

Note: * figure—percentage of the tree E—*Picea obovata* Ledeb., P—*Abies sibirica* Ledeb., B—*Betula pendula* L., Os—*Populus tremula* L.

**Table 2:** Tree condition category scale.

Tree Condition Category	External Signs	
	Coniferous	Deciduous
1—healthy (without signs of weakening)	the crown is thick; needles (foliage) are green; growth of the current year is normal	
2—weakened	the crown is sparse; needles are light green; growth is reduced but no more than by half; some branches are withered	the crown is sparse; foliage is light green; growth is reduced but no more than by half; some branches are withered; separate water shoots
3—severely weakened	the crown is open; needles are light green, matte; growth is weak, less than half of the usual; branches drying in 2/3 of the crown; fruit bodies of Polyporaceae fungi or the hollows typical of them; the initial stage of infection with bark beetles	the crown is open; foliage is small, light green; growth is weak, less than half of the usual; branches drying in 2/3 of the crown; abundant water shoots; fruit bodies of Polyporaceae fungi or the hollows typical of them
4—drying	the crown is strongly open; needles are gray, yellowish, or yellow-green; growth is very weak or absent; drying of more than 2/3 of the branches; mass infection with bark beetles, including in the lower part of the trunk	the crown is strongly open; foliage is small, sparse, light green, or yellowish; growth is very weak or absent; drying of more than 2/3 of the branches
5—dead	Trees that have completely lost their viability, including: the dead	

## Data Availability

The datasets generated and analyzed during the current study are available from the corresponding author on reasonable request.

## References

[1] Larionov M.V., Dogadina M.A., Tarakin A.V., Minakova I.V., Sentishcheva E.A., Bukreeva T.N. (2021). Creation of artificial phytocenoses with controlled properties as a tool for managing cultural ecosystems and landscapes. IOP Conf. Ser. Earth Environ. Sci..

[2] Rysin L.I., Saveliev L.I. (2002). Spruce Forests of Russia.

[3] Vasiljuskas V. Decline of Spruce Forest in Lithuania and Its Causes. Proceedings of the Problem of Spruce Forests Decline, Mogilev Belarus “Belforestprotection”.

[4] Hale S.E., Edwards C., Mason W.L., Price M.H. (2009). Relationships between canopy transmittance and stand parameters in Sitka spruce and Scots pine stands in Britain. Forestry.

[5] (2004). Eastern European Forests: History in the Holocene and the Present.

[6] (2008). Review of the Sanitary and Forest Pathological State of Russian Forests in 2006, 2007.

[7] Smith T.M., Reynolds R.W. (2003). Extended reconstruction of global sea surface temperatures based on COADS data (1854–1997). J. Clim..

[8] Brohan P., Kennedy J.J., Harris L., Tett S.F.B., Jones P.D. (2006). Uncertainty estimates in regional and global observed temperature changes: A new data set from 1850. J. Geophys. Res. Atmos..

[9] Odjugo P.A.O. (2010). Regional evidence of climate change in Nigeria. J. Geogr. Reg. Plan..

[10] Büntgen U., Krusic P.J., Piermattei A., David A.C., Jan E., Vladimir S.M., Alexander V.K., Camarero J.J., Crivellaro A., Körner C. (2019). Limited capacity of tree growth to mitigate the global greenhouse effect under predicted warming. Nat. Commun..

[11] Volodkin A.A., Volodkina O.A., Larionov M.V. (2002). Dynamics of reproduction of forest plantations in the forest-steppe zone of the Middle Volga Region. IOP Conf. Ser. Earth Environ. Sci..

[12] Din J., Khan S.U., Ali I., Gurmani A.R. (2011). Physiological and agronomic response of canola varieties to drought stress. J. Anim. Plant Sci..

[13] Ashraf M., Harris P.J.C. (2013). Photosynthesis under stressful environments: An overview. Photosynthetica.

[14] Bukharina I.L., Vedernikov K.E., Pashkova A.S. (2016). Morphophysiologic traits of spruce trees in conditions of Izhevsk. Contemp. Probl. Ecol..

[15] Volodkin A.A., Larionov M.V. (2021). Changes in the structure of forest communities in Penza region under the influence of natural factors. IOP Conf. Ser. Earth Environ. Sci..

[16] Anjum S.A., Xie X., Wang L. (2011). Morphological, physiological and biochemical responses of plants to drought stress. Afr. J. Agric. Res..

[17] Paul C., Brandl S., Friedrich S. (2019). Climate change and mixed forests: How do altered survival probabilities impact economically desirable species proportions of Norway spruce and European beech?. Ann. For. Sci..

[18] Piraino S. (2020). Assessing *Pinus pinea* L. resilience to three consecutive droughts in central-western Italian Peninsula. iFor.-Biogeosci. For..

[19] Honkaniemi J., Rammer W., Seidl R. (2020). Norway spruce at the trailing edge: The effect of landscape configuration and composition on climate resilience. Landsc. Ecol..

[20] Zamolodchikov D., Kraev G. (2016). Influence of Climate Change on the Forests of Russia: Recorded Impacts and Forecast Estimates. Sustain. For. Manag..

[21] Bukharina I.L., Povarnitsina T.M., Vedernikov K.E. (2007). Ecological and Biological Features of Woody Plants in an Urbanized Environment: Monograph.

[22] (1984). Taxation and Forest Management. The Growth of Wood in the Forest Stand. Classification and Symbolism, Basic Calculation Formulas. Terms and Definitions.

[23] Chertovskoy V.G. (1978). Spruce Forests of the European Part of the USSR.

[24] Vakhrushev K.V., Absalyamov R.R. (2017). Forestry complex of the Udmurt Republic: State, problems, prospects for the development of forest relations. Forests of Eurasia—Forests of the Volga Region, Proceedings of the XVII International Conference of Young Scientists Dedicated to the 150th Anniversary of the Birth of Professor G.F. Morozov, Kazan.

[25] Bukharina I.L., Pashkova A.S., Svetlakova O.A. Characteristic of the State of Spruce Plantations in the Area of Southern Taiga Forests of the Taiga Zone in the Udmurt Republic. Proceedings of the International Conference “Process Management and Scientific Developments”.

[26] Decree of the Head of the Udmurt Republic On Approval of the Forest Plan of the Udmurt Republic. 18 February 2019; No. 17. http://www.minpriroda.udm.ru/index.php?option=com_content&view=article&id=127&Itemid=234.

[27] Allen M.R., Babiker M., Chen Y. (2018). Summary for Policymakers: Special Report: Global Warming of 1.5 °C.

[28] Jandl R. (2020). Climate-Induced Challenges of Norway Spruce in Northern Austria.

[29] Bukharina I.L., Svetlakova O.A., Konopkova A., Ledneva O.S., Absalyamov R.R. (2019). Condition of forest litter in spruce plantations of the Republic of Udmurtia. AgroEkoInfo.

[30] (2014). A Brief Review of the Sanitary and Forest Pathological State of the Forests of the Udmurt Republic for 2013 and the Forecast of the Forest Pathological Situation for, 2014, 2013.

[31] Vedernikov K.E. (2012). Change in the Chemical Composition of Wood Picea obovata Ledeb. under the Influence of Ips typographus L. Khimiya Rastitel’nogoSyr’ya.

[32] Zhao T., Krokene P., Hu J., Christiansen E., Björklund N., Langstrom B., Solheim H., Borg-Karlson A.K. (2011). Induced terpene accumulation in Norway Spruceinhibits bark beetle colonization in a dose-dependent manner. PLoS ONE.

[33] Zhao T., Kandasamy D., Krokene P., Chen J., Gershenzon J., Hammerbacher A. (2019). Fungal associates of the tree-killing bark beetle, *Ips typographus,* vary in virulence, ability to degrade conifer phenolics and influence bark beetle tunneling behavior. Fungal Ecol..

[34] Konopkova A., Petek A., Kmet J. (2020). Impact of the European bark beetle *Ips typographus* on biochemical and growth properties of wood and needles in Siberian spruce *Picea obovata*. Cent. Eur. For. J..

[35] Bukharina I.L., Konopkova A., Vedernikov K.E. (2020). The state of the spruce stands of the boreal and boreal-subboreal forests of the eastern European plain in the territory of the Udmurt Republic (Russia). Ukr. J. Ecol..

[36] Vedernikov K., Zagrebin E., Bukharina I., Kuzmin P. (2022). Influence of the Biological and Chemical Structure of Spruce Wood on Xylophage Infestation. Florestae Ambiente.

[37] Thoss V., Byers J.A. (2006). Monoterpene chemo diversity of ponderosa pine in relation to herb ivory and bark beetle colonization. Chemoecology.

[38] Debkov N.M., Aleinikov A.A., Gradel A. (2019). Impacts of the invasive four eyed fir bark beetle (*Polygraphus proximus* Blandf.) on siberian fir (*Abies sibirica* Ledeb.) forests in southern Siberia. Geogr. Environ. Sustain..

[39] Maslov A.D. (2010). Bark-Beetle and Drying of Spruce Forests.

[40] Wermelinger B. (2004). Ecology and management of the spruce bark beetle *Ips typographus*—A review of recent research. For. Ecol. Manag..

[41] Larionov M.V., Volodkin A.A. (2021). Parameters of the state and biological stability of woody plants from native flora in conditions of artificial and natural phytocenoses. Nat. Tech. Sci..

[42] Larionov M.V., Volodkin A.A. (2022). Phytosozological study of natural monuments to determine the ecological status of specially protected natural areas. RUDN J. Ecol. Life Saf..

